# High-throughput mass spectrometry analysis revealed a role for glucosamine in potentiating recovery following desiccation stress in *Chironomus*

**DOI:** 10.1038/s41598-017-03572-5

**Published:** 2017-06-16

**Authors:** Leena Thorat, Dasharath Oulkar, Kaushik Banerjee, Sushama M. Gaikwad, Bimalendu B. Nath

**Affiliations:** 1 Stress Biology Research Laboratory, Department of Zoology, Savitribai Phule Pune University, Pune, 411007 India; 2 National Referral Laboratory, National Research Centre for Grapes, Pune, 412307 India; 3 Division of Biochemical Sciences, National Chemical Laboratory, Pune, 411008 India; 4 Department of Biotechnology, Savitribai Phule Pune University, Pune, 411007 India

## Abstract

Desiccation tolerance is an essential survival trait, especially in tropical aquatic organisms that are vulnerable to severe challenges posed by hydroperiodicity patterns in their habitats, characterized by dehydration-rehydration cycles. Here, we report a novel role for glucosamine as a desiccation stress-responsive metabolite in the underexplored tropical aquatic midge, *Chironomus ramosus*. Using high- throughput liquid chromatography quadrupole time-of-flight mass spectrometry (LC-QToF-MS) analysis, biochemical assays and gene expression studies, we confirmed that glucosamine was essential during the recovery phase in *C. ramosus* larvae. Additionally, we demonstrated that trehalose, a known stress-protectant was crucial during desiccation but did not offer any advantage to the larvae during recovery. Based on our findings, we emphasise on the collaborative interplay of glucosamine and trehalose in conferring overall resilience to desiccation stress and propose the involvement of the trehalose-chitin metabolic interface in insects as one of the stress-management strategies to potentiate recovery post desiccation through recruitment of glucosamine.

## Introduction

Fluctuations in ambient environmental conditions can trigger a vast array of adaptive strategies in organisms. In the purview of the current scenario of global climate change, desiccation happens to be one of the most environmentally relevant stressors that is crucial in determining the survival and distribution of all species^1–3^. Episodic temperature-humidity imbalances give rise to dehydration bouts posing organisms with severe challenges under physiological water deficits^4^. Under such circumstances, tolerance to desiccation can serve as a prime strategy, enabling organisms to sustain the dehydrated state in response to body water loss without compromising on the ability to revive and resume active metabolism upon return of favourable hydrating conditions^5–7^. The present study bears significance against the backdrop of the Intergovernmental Panel for Climate Change (IPCC)^8, 9^ which has raised concerns of the vulnerability of the entire biota including humans to the consequences of desiccation exposure owing to the risk of increasing rates in the frequency and severity of droughts in the coming years.

Since water acts as a limiting factor for aquatic ecosystems^10^, challenges and consequences of desiccation exposure can be severe for tropical ectotherms that experience dehydration-rehydration cycles on recurring basis in their aquatic habitats. Among aquatic ectotherms, chironomid midges form an abundant group of insects that play a key role in the maintenance of balanced ecosystems^11, 12^ and are capable of thriving under extreme environmental conditions^13, 14^. We have previously reported the impressive resilience of the larvae of a tropical midge species, *Chironomus ramosus* ^15, 16^ that are prone to daily fluctuations between wet and dry in their natural habitats (18.5551° N, 73.8618° E and 18.4818° N, 73.8296° E). We observed that natural populations of *C. ramosus* when subjected to transient desiccation bouts during tropical summers could revive following rainy season^15^. These observations provided us the impetus to use this underexplored species for the investigation of the desiccation physiology and biochemical strategies used by insects in general to combat dehydration stress. Being an understudied chironomid species, there is a dearth of information with regards to the desiccation biology of *C. ramosus* and hence the present study aims at the better understanding of its response to dehydration stress.

One of the predominant mechanisms of desiccation tolerance across various plant and animal taxa is the synthesis and accumulation of compatible solutes, mainly carbohydrates such as trehalose, mannose, sucrose, fructose, umbelliferose, mannitol, glycerol and sorbitol^17–25^. A vast diversity of Heat-Shock (HS) and Late Embryonic Abundant (LEA) proteins and amines such as proline and glycine-betaine have also been shown to play a critical role in desiccation tolerant organisms^26–30^. In addition, antioxidant enzymes and molecules have been identified for their protective role in organisms against the impact of ionic disequilibrium in response to desiccation-mediated oxidative stress^31–35^. Very recently, the “omics” methodology is being increasingly employed for the understanding of regulatory mechanisms through transcriptome and proteome investigations and bioinformatics analyses that underlie the molecular physiology associated with desiccation tolerance^36^. These investigations have provided useful insights towards understanding of the survival basis of organisms in dehydrating environments and its association with the organismal physiology and population dynamics.

In the context of aquatic desiccation tolerant insects in particular, the chironomid midge *Polypedilum vanderplanki* has been extensively studied for the molecular, biochemical and physiological mechanisms governing its extreme ability to tolerate water loss^37^. In fact, to date, *P. vanderplanki* is the largest known eukaryote that can endure nearly 97% water loss followed by entry into an ametabolic state, termed ‘anhydrobiosis’ which can be sustained for 17 years until rehydration^38, 39^. Comparative genomics of *P. vanderplanki* and another related but desiccation-sensitive species *Polypedilum nubifer* has indicated that specific genomic signatures present in *P. vanderplanki* (but absent in *P. nubifer*) contribute to its exceptional anhydrobiotic potential^40^. Recent work from our laboratory in the tropical midge, *C*. *ramosus* has shown that the larvae have the ability to recover following desiccation exposure under laboratory conditions^15^. However, it must be noted that *C. ramous* exhibits low ability to tolerate water loss and is hence termed desiccation tolerant unlike *P. vanderplanki* which is highly anhydrobiotic. Furthermore, we have shown that desiccation tolerance in *C. ramosus* is associated with developmental trade-offs^15^. Here we extend our investigation towards the identification of compatible solutes using high-throughput UPLC-LC-QToF-MS analysis, enzyme activity assays and gene expression studies that were correlated with revival and survival post desiccation. With the identification of glucosamine as a novel desiccation stress-responsive metabolite, we report a new candidate biomolecule in the desiccation physiology of insects. In addition, we also demonstrated the critical role of trehalose, a well-established stress protectant which collaborates with glucosamine in larval desiccation tolerance in *C. ramosus*. Based on these findings, we propose the trehalose-chitin metabolic interface in insects as one of the stress-management strategies to potentiate recovery from desiccation through recruitment of glucosamine.

## Results and Discussion

### Desiccation tolerance threshold

**Figure 1:**
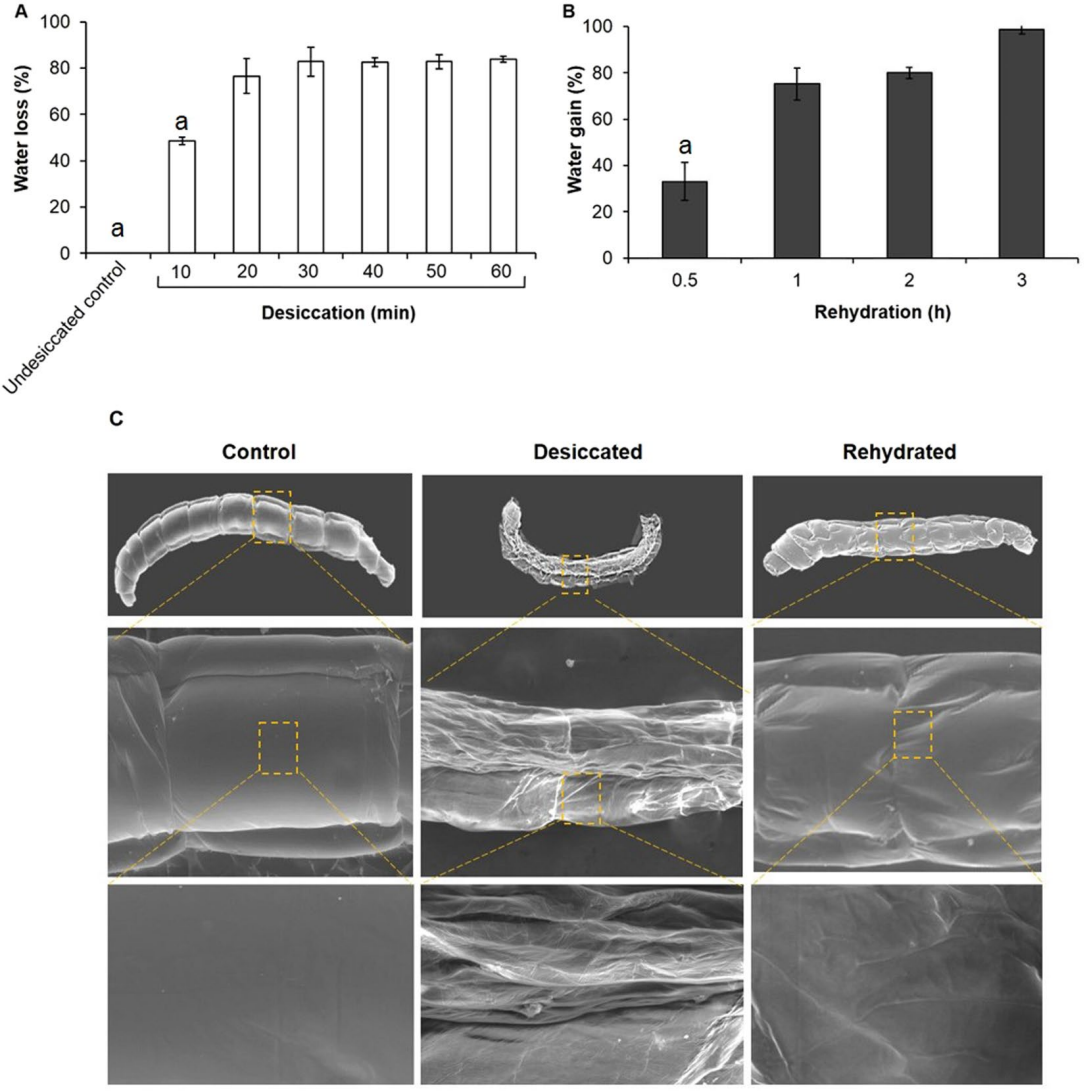
Overview of desiccation stress response in *C. ramosus* larvae. (**A**) Water loss during progressive desiccation. Data represented are mean ± SD of three replicates (a = p < 0.05 vs 40 min desiccation). (**B**) Water intake upon rehydration. Data represented are mean ± SD of three replicates (a = p < 0.05 vs 3 h rehydration). (**C**) Representative E-SEM photomicrographs of control, desiccated and rehydrated larvae showing surface topology of whole larva (top panel; x30), segment of the larval body (middle panel; x250) and view of outer integument (last panel; x1000).

We tested desiccation tolerance threshold of the larvae i.e. ability to withstand desiccation exposure under laboratory conditions at 3–5% relative humidity for 1 h. Larval desiccation tolerance threshold was found to be 50 ± 10 min during which rapid loss of body water occurred with progressive desiccation (Fig. 1A) Rehydration reversed the situation with water intake occurring at a very fast rate (Fig. 1B). The initial slow body movements and the characteristic larval undulations became subsequently prominent as recovery progressed, thereby confirming the revival ability of the larvae. We observed 56 ± 7% larval recovery upon rehydration, of which, ~40 ± 2% larvae metamorphosed into adults while the remaining died at pupation. Preliminary assessment by Environmental-Scanning Electron Microscopy (E-SEM) revealed cuticular shrinkage which was not surprising, given the amount of body water loss suffered by the larvae (Fig. 1C). As evident, we observed remarkable restoration of the shrunken cuticle and characteristic larval movements upon rehydration (Supplementary Movie S1). With progressive rehydration, larval movements became more prominent, characterised by increased frequency of wriggling movements. This characteristic ‘undulatory movement’ was considered as a behavioural parameter for judging larval recovery following desiccation stress (details furnished in materials and methods). Furthermore, to ascertain the plausible cause of rapid water loss in the larvae, we performed histochemical examinations of hematoxylin-eosin stained larval body integument. For comparison, we carried out a parallel study in the terrestrial dipteran insect, *Drosophila melanogaster* which has been shown to lose body water gradually under desiccation stress over a period of 10 ± 0.45 h^22^. We found that the outer body integument of *C. ramosus* measured 1.4 ± 0.01 mm in thickness while that of *D. melanogaster* averaged to 2.6 ± 0.12 mm, which was almost twice as that of *C. ramosus* (Fig. S1). Thus, the comparatively thinner outer body integument of *C. ramosus* can be correlated to its vulnerability for quick loss of water during acute desiccation exposure with an overall tolerance of only 50 ± 10 min as compared to that of *D. melanogaster*. Similar interpretations have been drawn from the comparative cuticular waterproofing ability in the chironomids, *P. vanderplanki* and *Paraborniella tonnoiri* resulting in differential drought-combating strategies in these species which have been correlated with their contrasting habitats^41^.

### Detection of candidate biomolecules

We next sought to identify the putative biomolecules that might be crucial for desiccation tolerance in the midge larvae. A full scan screening approach performed on an Acquity UPLC, coupled to Q-ToF-MS, revealed positive identification of the metabolites which was confirmed based on <5 ppm mass accuracy of precursor along with at least one diagnostic product ions (with 5 ppm). QToF-MS was operated with electrospray ionisation (ESI) in nominal resolution 20000, positive polarity and controlled by MassLynx 4.1. We identified trehalose and glucosamine as the desiccation stress-responsive biomolecules that enhanced endurance of the larvae under unavailability of metabolic water. Although trehalose happens to be one of the most extensively occurring stress protectants in animals^39–41^, glucosamine has never been reported before for its protective role under stressful environments, including desiccation. However, glucosamine, has been implicated in stability of cell membrane^42^ and has been in use for its therapeutic properties^43^.

### Trehalose

**Figure 2:**
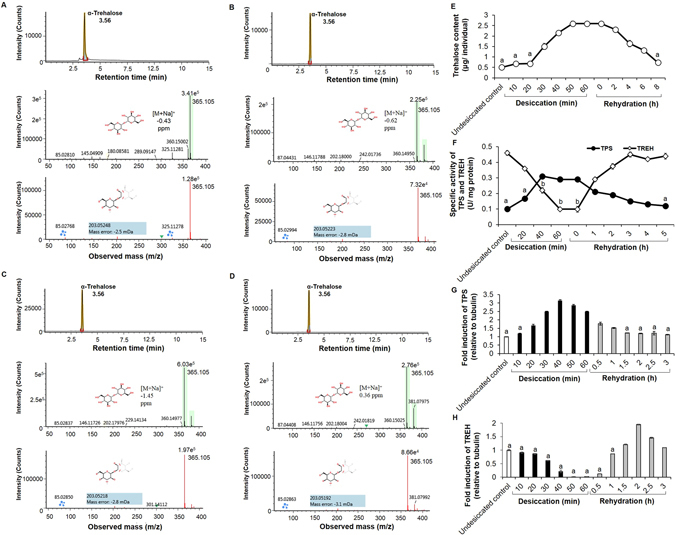
Desiccation-induced trehalose synthesis in the larvae. (**A–D**) Representative UPLC-QToF-MS chromatograms indicating the detection of trehalose in the midge larvae. Trehalose was identified as [M + Na]^+^ adduct with mass accuracy −0.43 ppm with confirmatory ions (203.05248 Da, 145.04906 Da and 85.02768 Da). (**A**) Standard trehalose solution. (**B**) Undesiccated control (**C**) 50 min desiccation. (**D**) 5 h rehydration. (**E**) High trehalose content was prominent during desiccation exposure followed by rapid decline upon rehydration. Data represented are mean ± SD of three replicates (a = p < 0.05 vs 50 min desiccation). (**F**) TPS and TREH enzyme activities (U/mg protein) during desiccation and rehydration. Data represented are mean ± SD of three replicates (a = p < 0.05 vs 40 min desiccation for TPS; b = p < 0.05 vs 3 h rehydration for TREH). (**G**) Fold induction of tps transcripts during desiccation and rehydration. Data represented are mean ± SD of three replicates (a = p < 0.05 vs 40 min desiccation). Undesiccated control values were set to 1 against which all other values were compared. The results were represented as fold induction relative to tubulin. (**H**) Fold induction of treh transcripts during desiccation and rehydration. Data represented are mean ± SD of three replicates (a = p < 0.05 vs 2 h rehydration). Undesiccated control values were set to 1 against which all other values were compared. The results were represented as fold induction relative to tubulin.

With reference to trehalose, we found approximately 4-fold excess within 50 ± 10 min of desiccation exposure as compared to the undesiccated control group (Fig. 2A–E). Trehalose (m/z = 365.10527 Da) was identified as [M+Na]^+^ adduct with mass accuracy −0.43 ppm with confirmatory ions (203.05248 Da, 145.04906 Da and 85.02768 Da) (Fig. 2A–D). Rehydration on the other hand, ensured hydrolysis of the accumulated trehalose, thereby restoring basal levels in the larvae (Fig. 2E, Table S1). We^31^ and others^44^ have previously demonstrated that exogenous trehalose supplementation in insects improved endurance to desiccation stress. On similar lines, in this study, we wished to assess if exogenous trehalose feeding prior to desiccation could contribute to desiccation tolerance in the larvae. Trehalose transporter in insects carries out the bidirectional transport of trehalose from the fat body and gut across other tissues^45, 46^. Thus, we speculated that exogenous trehalose fed to the larvae was transported to the hemolymph *via* the trehalose transporter from the gut. We found that *Chironomus* larvae fed with trehalose prior to desiccation (Fig. S2) showed marked improvement in threshold desiccation tolerance (Fig. S3A,B). However, trehalose feeding prior to rehydration (Fig. S4) did not offer any apparent benefit to the larvae in terms of recovery (Fig. S5A,B). To further understand the importance of desiccation-induced trehalose metabolism, we determined the activity of key enzymes involved in trehalose metabolism. Insect trehalose synthesis (Fig. S6) is governed by trehalose 6-phosphate synthase (TPS; EC 2.4.1.15) while trehalase (TREH; EC 3.2.1.28) is responsible for its degradation^47^. Quite obviously, we found increased TPS activity as desiccation progressed, which further decreased post rehydration (Fig. 2F). These data corroborated with quantitative RT-PCR results which indicated corresponding increment and decrement in tps transcript levels (Fig. 2G). In contrast, activity of TREH and levels of treh transcripts were seen to decline during desiccation followed by a rapid increase during the recovery phase (Fig. 2F,H). Although trehalose accumulation in the desiccated larvae did not occur in a dramatic manner as compared to the undesiccated controls, nevertheless, the fact that trehalose-fed larvae show improved tolerance to desiccation clearly suggested an essential role for trehalose in desiccation stress response in *C. ramosus* larvae. Similar lack of drastic accumulation or absence of trehalose in other anhydrobiotes has been reinforced from findings suggesting that presence of trehalose or non-reducing disaccharides cannot be considered as a universal requirement and organisms do adopt alternate survival strategies such as recruitment of hydrophilic proteins, LEA and HSPs that could be more crucial compared to mere carbohydrate pools^48–50^. These observations provide insights into the diversity in invertebrate desiccation tolerance from ecological, physiological, biochemical, molecular and evolutionary perspectives. The exploration of other desiccation-induced biomolecules was out of scope of the present study, however, their involvement cannot be ruled out. Nevertheless, we have previously reported the role of Hsp70 during larval desiccation tolerance in *C. ramosus* ^15^.

### Glucosamine

**Figure 3:**
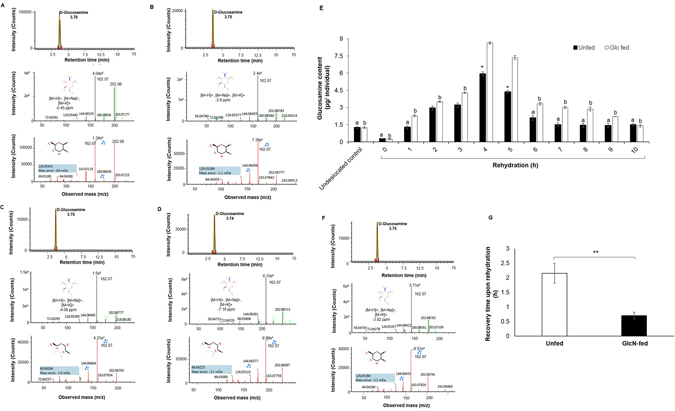
Discovery of glucosamine and the biosynthetic pathway followed during recovery post desiccation. (**A–D**) Representative UPLC-QToF-MS chromatograms indicating the detection of glucosamine. Glucosamine was identified as [M+H]^+^, [M+Na]^+^, [M+K]^+^ with mass accuracy 0.45 ppm along with the confirmatory ions (162.07532 Da, 126.05370 Da and 84.04294 Da). (**A**) Standard glucosamine solution. (**B**) Undesiccated control. (**C**) 50 min desiccation. (**D**) 6 h rehydration. (**E**) Comparative glucosamine content in the fed and unfed larvae during rehydration. Of note, glucosamine levels were significantly higher in the fed group as compared to unfed between 4 and 5 h rehydration (*p < 0.05). Data represented are mean ± SD of three replicates (a = p < 0.05 vs 4 h rehydration in GlcN-unfed group; b = p < 0.05 vs 4 h rehydration in GlcN-fed group). (**F**) Representative UPLC-QToF-MS chromatograms indicating exceptionally high glucosamine signal in Glc-fed larvae at 6 h rehydration, suggesting the accumulation of exogenously fed + endogenous glucosamine in the larvae. (**G**) Glucosamine fed larvae recovered faster upon rehydration in comparison to the unfed larvae. Data represented are mean ± SD of three replicates. **p < 0.05, Student’s *t*-test.

The most intriguing finding was the discovery of glucosamine as a stress-alleviating metabolite in the chironomid larvae (Fig. 3A–G) wherein larvae harboured nearly 4-fold excess of glucosamine pools during the rehydration phase relative to their corresponding undesiccated controls. UPLC-QToF-MS results revealed the presence of glucosamine (m/z = 180.0861 Da) with the formation of three different adducts *viz*. [M+H]^+^, [M+Na]^+^, [M+K]^+^. Of these, [M+Na]^+^ was prominent with mass accuracy 0.45 ppm along with confirmatory ions (162.07532 Da, 126.05370 Da and 84.04294 Da) (Fig. 3A–D). Since relatively high glucosamine signal appeared only during rehydration (Fig. 3E, Table S2), it was therefore of interest to examine if exogenously fed glucosamine could provide any added advantage for recovery to the larvae. To explore this possibility, we fed the desiccated larvae either with glucosamine solution (GlcN-fed group) or with water (non-fed group) prior to rehydration for 1 h. Glucose transporters are uniporters showing varying degrees of specificity towards glucose or other hexoses without any absolute specificity for individual sugars and GLUT2, a glucose transporter is known to transport glucosamine with high affinity^51^. A GLUT2-like transporter has been shown to be present in the midgut cells of the insect larvae, *Aphidius ervi* that transports glucosamine across other body tissues^52^. In a similar fashion, we reasoned that exogenously fed glucosamine in *Chironomus* larvae was transported to the hemolymph and other tissues *via* the transporter from the gut. We confirmed the uptake of exogenously fed GlcN by the GlcN-fed larvae prior to rehydration (Fig. S7), wherein Glc-fed larvae showed ~4 fold excess of GlcN content compared to the undesiccated control and the unfed larvae (Fig. 3B,C). The considerably high GlcN levels in the GlcN-fed larvae (Fig. 3F) contributed to the apparent enhancement in larval recovery (within 0.7 ± 0.12 h) (Fig. 3G). On the other hand, the unfed larvae needed some more time in order to attain required GlcN levels. In the unfed larvae, GlcN levels started rising between 1 and 2 h of recovery (Fig. 3E), during which, larvae also began to revive (within 2.15 ± 0.34 h- Fig. 3G). Based on these observations, we attribute the role of GlcN in larval recovery from desiccation stress. This was in contrast with the case observed in the larvae fed with trehalose prior to rehydration wherein trehalose apparently did not offer any benefit to the larvae in terms of revival upon rehydration (refer to Fig. S5). Furthermore, we also found that glucosamine pools in the larvae were maximum during 4 and 5 h rehydration and GlcN-fed larvae indeed possessed almost 6-fold excess glucosamine content (endogenous + exogenously fed) as compared to the undesiccated control larvae (Fig. 3E).

### Desiccation-induced involvement of chitin metabolic pathway

The above observations raised further questions of the role of glucosamine in larval desiccation tolerance. Glucosamine is central to the chitin metabolic pathway in insects^53^ and hence we found it worthwhile to assess the possible involvement of glucosamine in desiccation-mediated chitin metabolism events in the larvae.

### Chitin deacetylase

**Figure 4:**
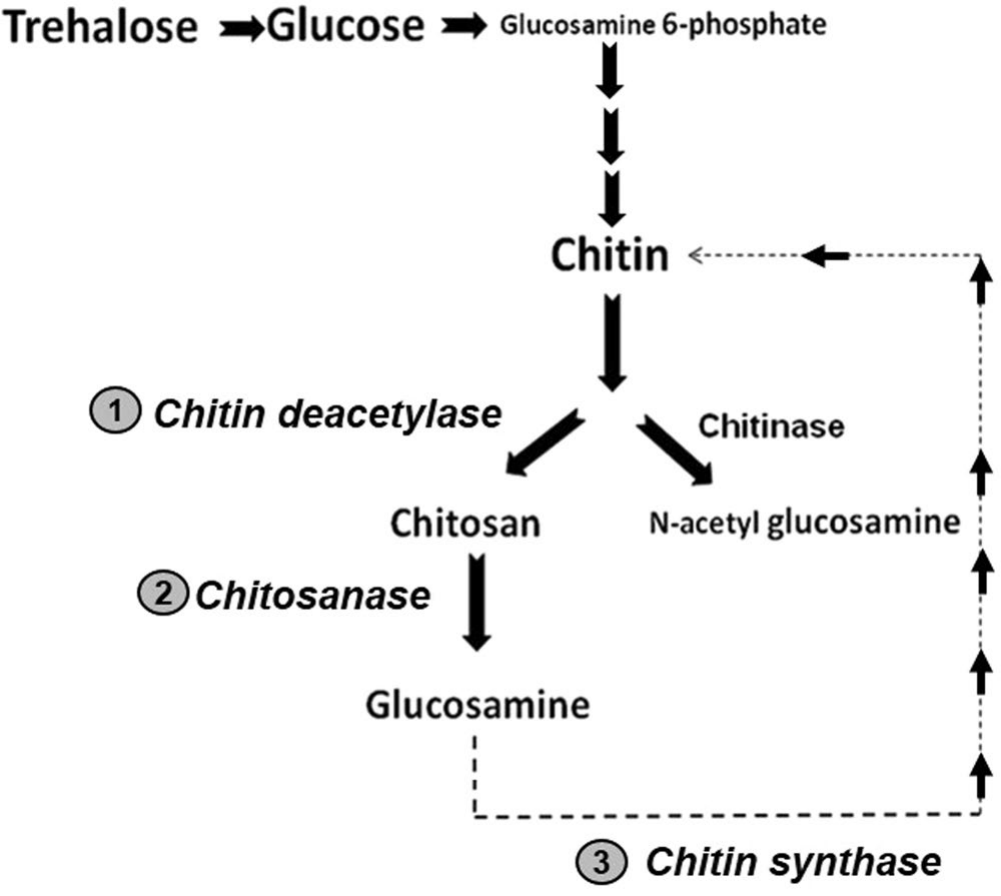
Cascade of events involved in chitin metabolism in *C*. *ramosus*. In response to desiccation, *C. ramosus* followed the Chitin deacetylase (CDA) pathway for chitin degradation (step 1). Further, chitosanase generated glucosamine (step 2) which was utilized for restoration of the desiccated cuticle by chitin synthase (step 3).

**Figure 5:**
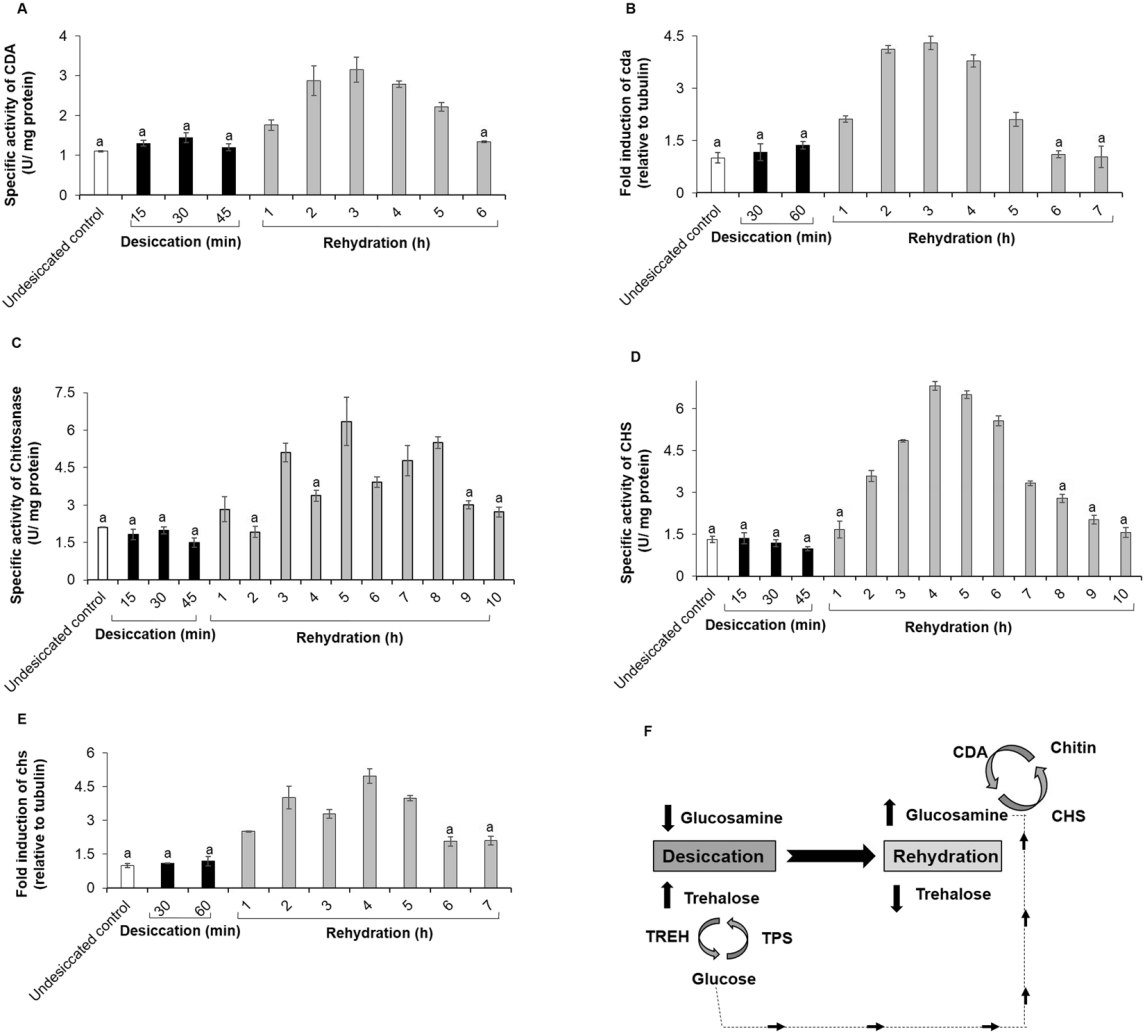
Involvement of the chitin metabolic pathway and working model underlying the basis of desiccation tolerance. (**A**) CDA enzyme activity. Data represented are mean ± SD of eight replicates (a = p < 0.05 vs 3 h rehydration). (**B**) Fold induction of cda transcripts. Data represented are mean ± SD of eight replicates (a = p < 0.05 vs 3 h rehydration). Undesiccated control values were set to 1 against which all other values were compared. The results were represented as fold induction relative to tubulin. (**C**) Chitosanase enzyme activity. Data represented are mean ± SD of eight replicates (a = p < 0.05 vs 5 h rehydration). (**D**) Chitin synthase enzyme activity. Data represented are mean ± SD of eight replicates (a = p < 0.05 vs 4 h rehydration). (**E**) Fold induction of chs transcripts. Data represented are mean ± SD of eight replicates (a = p < 0.05 vs 4 h rehydration). Undesiccated control values were set to 1 against which all other values were compared. The results were represented as fold induction relative to tubulin. (**F**) Schematic depicting the collaborative interplay of key biomolecular players namely, trehalose, chitin and glucosamine implicated during desiccation tolerance in *C. ramosus*.

Chitin is an abundant natural polymer composed of N-acetylglucosamine found in insect cuticle, crustacean shells, molluscan skeletons and fungi^54^. It is generally well-accepted that chitin degradation can be driven in two ways^55^: either by the action of chitinase (CHT; EC 3.2.1.14) which generates N-acetyl glucosamine monomers or by chitin deacetylase (CDA; EC 3.5.1.41) which yields glucosamine *via* an intermediate step (Fig. 4A). In our case, UPLC-(ESI)-QToF-MS data showed the presence of glucosamine (and not N-acetyl glucosamine- Fig. S8, Table S3). We therefore speculated that midge larvae followed the CDA pathway resulting in the generation of glucosamine. In agreement to this view, we observed an increment in the CDA activity beginning from 1 h rehydration that continued to rise up to 3 h and maintained a relatively high level until 5 h, thereafter declining to basal levels (Fig. 4-step 1, Fig. 5A and Fig. S9). Quantitative RT-PCR results indicated elevated levels of Cda during the early hours of rehydration (Fig. 5B). CDA is responsible for the degradation of chitin into chitosan which is further used as substrate by chitosanase enzyme (EC 3.2.1.132) (Fig. 4-step 2 and Fig. 5C) for the generation of glucosamine. Increasing CDA levels (Fig. 5A, Fig. S9) triggered the subsequent rise in chitosanase enzyme activity up to 8 h with intermittent rise starting from 1 h hour rehydration (Fig. 5C) which resulted in subsequent rise in glucosamine levels (Fig. 4-step 2, Fig. 3E and Fig. S9).

### Chitin synthase

Having confirmed the source of glucosamine generation, we further sought to understand its putative role in larval desiccation tolerance. Vulnerability of cuticle during desiccation stress increases because chitin is the first portal of water loss in insects. Moreover, previous studies have shown that insects possess the ability to recycle chitin degradation products in response to environmental assaults^53–55^. Likewise, a similar situation can be expected in our case where the shrunken larval cuticle could be recycled for the restoration of cuticular integrity. We therefore targeted to evaluate chitin synthase (CHS; EC 2.3.1.16) enzyme which was found to be activated (Fig. 4-step 3, Fig. 5D and Fig. S9) almost concomitantly following chitosanase enzyme activity (Fig. 5C). We also observed elevation of chs transcript from 1 h post rehydration (Fig. 5E) followed by increment in CHS enzyme activity. As compared to the undesiccated control, rising CHS activity was substantially high during 3 and 6 h of rehydration. As seen in Fig. 3E, beyond 5 h rehydration, glucosamine levels started declining, indicative of the utilisation of glucosamine by CHS. Thus, consistent with our assumption, the coordinated temporal regulation of CDA followed by chitosanase and CHS (Fig. S9) was important for the amelioration of larval recovery process supposedly by the recruitment of glucosamine molecules. Taken together, glucosamine feeding allowed us to directly assess its significance from the functional standpoint in potentiating recovery following desiccation stress in the larvae.

Chitin metabolism is a dynamic process in insects. Chitin is known to constitute ~1–4% of the total fresh weight of insects^56^ and of this amount, ~50% is lost as exuviae. At any given time prior to adulthood, ~2% chitin is referred to as chitin in flux (characterized either by chitin synthesis or chitin degradation)^56^. In other words, constitutive presence of key metabolites of chitin is obvious in insects. Moreover, as insects can recycle chitin under stress conditions using chitin degradation products^53–55^, the quick turnover of chitin-related metabolites seen in our study was logically expected. As a consequence, increased basal levels of the components in the chitin metabolic pathway were found during the recovery phase, given the body’s need for the restoration of cuticular integrity.

### Working hypothesis: trehalose-chitin metabolic interface

Balanced environmental parameters such as temperature coupled with humidity play a crucial role in body water homeostasis in organisms^57–60^ and few animals, plants and fungi possess the ability to withstand the dehydrated state under depleted water levels^61–64^. Here, we have discussed the biochemical intricacies involving the activation of key physiological mechanisms that confer tolerance to insects under desiccation exposure using *C. ramosus* larvae. The metabolic route of glucosamine generation *via* CDA pathway in the recovering midge larvae supposedly contributed to the sturdiness of the chitinous exoskeleton which is an immediate requirement during the stress-recovery phase. Our findings reflect the rationale of Zhu *et al*.^65^, suggesting that CDA could play a vital role in a variety of insect species with respect to deacetylation of chitin, thereby making it more resistant to hydrolysis by endochitinases. In the light of the above findings, we propose a working model that establishes a link between trehalose and glucosamine metabolism (Fig. 5F). To sum up, chitin metabolism in insects essentially begins from trehalose (Fig. S10). Glucose liberated upon trehalose hydrolysis is utilized as raw material to produce chitin following a series of steps. Chitin degradation generates glucosamine which in turn is utilized for the restoration of the desiccated cuticle (Fig. 5F, Fig. S10). Thus, we propose a functional significance of the trehalose-chitin metabolic interface in alleviating desiccation stress in *C. ramosus* larvae.

A wealth of information is available on the biochemical, behavioural and molecular underpinnings in desiccation tolerant animals such as the brine shrimps^66–68^, tardigrades^69–71^, rotifers^48, 49, 72^, nematodes^73–76^ and extremophilic midge species including the rockpool dwelling *P. vanderplanki* ^39, 40, 77^ and the Antarctic midge, *Belgica antarctica* ^78–80^ that have been studied elaborately for their unique repertoire of molecular and biochemical signatures which confer them with anhydrobiotic ability. It must be noted that a commonality of desiccation tolerance mechanisms in most organisms studied till date is the desiccation protective role of trehalose; however, to the best of the authors’ knowledge, evidences supporting the stress protectant role of glucosamine have not been proposed till date. The present study demonstrated that the desiccation tolerance threshold of *C. ramosus* larvae under desiccation exposure was 50 ± 10 min and that the larvae could revive only if rehydrated immediately. Thus *C. ramosus* possesses a considerably lower degree of desiccation tolerance in comparison to the anhydrobiotic chironomid, *P. vanderplanki* ^39^. Since desiccation stress response patterns are known to vary among species depending on their ecological habitats and the rate and mode of dehydration^81^, one can expect a wide spectrum of species-level variations in the physiological adaptations towards survival^82^. In the case of chironomid midges, for instance, it has been showed that ecological niche is a key determinant of desiccation stress response among the twelve Japanese chironomid species tested in a study^83^. As an extension of this work, our laboratory developed a quantitative estimation tool, termed the ‘desiccation tolerance index’ to validate the correlation between desiccation tolerance and geographic distributional patterns of Oriental chironomids including *C. ramosus* (which showed a relatively low tolerance index)^16^. Given its extreme anhydrobiotic potential, *P. vanderplanki* is bound to be at the top-most position on this scale among the existing desiccation tolerant chironomids known till date.

It is interesting to note that the tremendous demand for biochemical and physiological changes in *C. ramosus* larvae leading to quick induction of gene regulation resulted in trade-offs such as inability to revive if not rehydrated immediately as shown in this study and desiccation-induced developmental heterochrony^15^. Sudden stress exposure requires rapid adaptation and tremendous reorganization of body physiology^84^. In this context, immediate-early genes (IEGs) are known to get activated and transcribed within minutes of intrinsic or extrinsic stimulation without requiring *de novo* protein synthesis^85^. Classical examples of regulation of IEGs that are elicited almost instantaneously or within few seconds or minutes of stress treatment include the Heat-shock genes^86–89^ and the hypoxia inducible factor (HIF) signaling^90, 91^. A similar case of drought-responsive quick gene induction (within 20 min) was demonstrated in *Arabidopsis thaliana* ^92^ and in the midge *Chironomus striatipennis* (within 60 min) under heat shock^93^. Such rapid response mechanisms are believed to be achieved by modifying general parameters of translation, namely, reduced stringency of start codon selection and increased accuracy of translation termination^85, 94^.

## Conclusions

The family Chironomidae forms a large group of aquatic macroinvertebrates that rely on temperature-humidity balance in their freshwater ecosystems, thereby serving as a convenient and useful representative insect for the assessment of tolerance patterns of environmentally relevant stressors such as desiccation. Here, we explored one of the physiological mechanisms of desiccation tolerance in *Chironomus ramosus*, an understudied tropical midge that is prone to recurrent hydroperiodicity fluctuations in its habitats. Major highlights of our work are: (i) it confirms a novel role for glucosamine as a desiccation stress-responsive biomolecule that is crucial for recovery in *C. ramosus* larvae, (ii) it demonstrates that trehalose is required by the larvae for sustenance in the desiccated state but is not involved during the recovery phase and (iii) it emphasises on the collaborative interplay of glucosamine and trehalose in conferring overall resilience to desiccation stress in the larvae, thereby proposing the involvement of the trehalose-chitin metabolic interface in insects as one of the stress-management strategies to potentiate recovery post desiccation.

## Materials and Methods

### Chemicals

Unless stated otherwise, all chemicals were obtained from Sigma-Aldrich (USA).

### Organisms

Isofemale lines of *C. ramosus* were maintained under controlled laboratory conditions at 25 ± 1 °C and 14 h L: 10 h D. Larvae were supplemented with food medium as described before^95^. Third instar larvae from inbred populations of *C. ramosus* were used for all further experiments.

### Desiccation treatment with and without trehalose

Larvae were given a brief rinse in water to clean any debri surrounding the larval body. Prior to desiccation, one batch of larvae were incubated in a glass vial in 5 ml of 25 mM trehalose (standardized effective dose) solution for 1 h as described before^31^. These larvae are henceforth referred to as the trehalose-fed group. Parallelly, another batch of larvae were incubated in a glass vial in 5 ml water for 1 h and were referred to as the unfed larvae. After incubation in water or trehalose, larvae were given a quick rinse in water to ensure the removal of traces of trehalose solution on the larval body. Larvae were then transferred to Whatman filter paper to remove excess moisture adhering to the body and gently picked up using a brush and placed in a desiccator on a Petri dish lined with dry tissue paper. 10 μl of water was given to each larva in order to prevent drying during the process of placing the larva inside the desiccator. Larvae were then allowed to dehydrate for 1 h inside the desiccator maintained at 3–5% relative humidity and 23 ± 1 °C. Undesiccated larvae were used as controls.

### Rehydration with and without trehalose and glucosamine feeding

Desiccated larvae were gently picked up using a brush and incubated in glass vials containing either in 5 ml of 25 mM trehalose (standardized effective dose) solution or 5 ml of 55 mM glucosamine (standardized effective dose) solution for 1 h (henceforth referred to trehalose-fed and GlcN-fed larvae respectively). Larvae incubated in water for 1 h constituted the unfed larval group. After incubation in trehalose or GlcN or water, all the three larvae groups were allowed to rehydrate in water only, under ambient conditions for recovery. Initial slight body movements characterized by ‘jerks’ and ‘wriggling’ were considered as the early signs of revival. Larval survival was judged by gentle stimulation with a non-abrasive brush. With progressive rehydration, larval movements became more prominent and were now characterised by increased frequency of wriggling movements defined as ‘undulatory movement’ which was used as a behavioural parameter for judging larval recovery after desiccation stress. One larval undulation was considered as one complete wave-like motion, starting from the head and traversing in an antero-posterior direction which ends at the tip of the posterior parapods^96^. We defined the beginning of this undulatory movement as larval recovery. Dead larvae were removed prior to all analyses to rule out the possible contribution of metabolites from the dead larvae. Videos were captured on Dino-Lite microscope (AD 4013 T).

### Gravimetric analysis

Water content in terms of water loss and gain was assessed according to published methods^97^. Larvae were weighed in groups of 30 before desiccation exposure and at every 15 minute intervals during progressive desiccation until 1 hour. Similarly, weight measurements were performed at various time intervals during rehydration.

### Microscopy

Environmental-Scanning Electron Microscopy (E-SEM) was conducted on samples of control, dehydrated and rehydrated larvae on FEI Quanta 200 3D system, The Netherlands. Specimens were directly mounted on Peltier cooling stubs covered with carbon black tape. E-SEM was carried out without any experimental procedures unlike the conventional SEM protocol. Analysis was carried out on low vacuum mode and low accelerating voltage at 20 kV. All micrographs were captured using xT Microscope Server (version 1.7.3).

### UPLC-(ESI)-QToF-MS

Groups of 30 larvae were picked up at appropriate time intervals for sample preparation. Larvae were homogenized in 300 µl of 90% ethanol and centrifuged at 1500 g for 10 min. Supernatants obtained were transferred to fresh centrifuge tubes and heated on a water bath for evaporation until dry. The precipitate was reconstituted in 1:1 methanol-water and preserved at −20 °C until use. Identification of metabolites was carried out on an Acquity UPLC, coupled to QToF-MS (Synapt G2 HDMS, Waters Corporation, Manchester, UK). QToF-MS was operated with electrospray ionisation (ESI) in nominal resolution 20,000 and controlled by MassLynx 4.1. Data acquisition was performed with MSE function with continuum mode in the range of m/z 50–600. The MSE mode provided a full scan MS data (low energy, 4 V) and MS/MS data (high energy, 10–60 V ramping) simultaneously. The source parameters were set as follows: capillary 3 kV, sampling cone 30 V, extraction cone 5 V, source temperature 120 °C, desolvation temperature 500 °C, desolvation gas flow 1000 l/h and cone gas 50 L/h. For mass spectrometer calibration, 0.5 mM sodium formate was used. The lock spray reference mass Leucine Enkephalin (m/z 556.2771 in positive polarity) was used for mass correction with a flow rate 10 µl/min and concentration of 2 µg/ml at 20 sec interval. Chromatographic separation was performed on XBridge HILIC column 2.1 × 100 mm, 3 µm (Waters India Pvt. Ltd., Bangalore, India) at 35 °C. The mobile phase consisted of A phase: water (20 mM Ammonium formate) and B phase acetonitrile used in gradient program with 0.4 ml/min flow rate. The gradient program was 0–1 min 100% B, 4.5 min 100–40% B, 5–8 min 40%B, 8–9 min 40–100% B, 9–15 min 100% B. The injection volume was 5 µl and the samples were kept at 15 °C throughout the analysis. Since the analytes were highly polar, retention on the C18 column was very poor hence hydrophilic interaction liquid chromatography (HILIC) column was selected which provided better retention and peak shape for all three analytes. Raw data acquired for all the standard solutions and experimental samples were processed with the help of UNIFI software 1.7 version (Waters Corporation, Manchester, UK).

### Enzyme assays

All enzyme assays were carried out on a UV-Vis spectrophotometer (V-630; JASCO International, Japan) and protein measurements and preparation of larval extracts for all assays were performed as follows.

#### Enzymes involved in trehalose metabolism

Trehalose 6-phosphate synthase (TPS) and trehalase (TREH) enzyme activities were evaluated in the desiccating and rehydrating larvae as previously described earlier^22^. Spectrophotometric measurements were recorded at 340 nm and enzyme activities were calculated as units per mg protein (U/mg protein).

#### Enzymes involved in chitin metabolism

Chitin deacetylase (CDA) enzyme assay was carried out in two parts. First, commercial chitin was treated with larval extract to generate the deacetylated substrate, chitosan along with acetate^98^. Next, quantification of acetate residues was carried out by standard assay procedure (K-ACET 02/11; Megazyme assay kit, Wicklow, Ireland). The amount of acetate residues generated was used as a measure of chitin deacetylation by CDA. Spectrophotometric measurements were recorded at 340 nm and CDA activity was calculated (U/mg protein).

Chitosanase assay was performed following published protocol^99^. Larval extracts were used for the estimation of reducing sugars produced from chitosan Absorbance was measured at 590 nm and chitosanase activity (U/mg protein) was determined.

Chitin synthase (CHS) assay was based on a published protocol^100^ with minor modifications. Optical density (403 nm) was determined on an ELISA Reader (BIORAD; Microplate Reader 680) and CHS activity was estimated (nmolGlcNAc.mg^−1^. hour^−1^) and expressed as U/mg protein.

### RNA isolation and cDNA synthesis

Larvae were picked up at appropriate desiccation and recovery time points and frozen down in liquid nitrogen. Total RNA isolation was carried out as per manufacturer’s instructions using the peqGOLD Total RNA Kit (Germany) including DNAse I digestion step as previously described^15^. 1 μg of the total eluted RNA was used for the synthesis of first-strand cDNA following manufacturer’s instructions (M-MLV Reverse Transcriptase Kit; Invitrogen).

### Reverse transcription quantitative PCR (RTqPCR) analysis

First-strand cDNA (1:20 dilution) was used as a template to quantify transcripts of the genes of interest by RT-qPCR analysis on Step One System (Applied Biosystems, USA) using appropriate primer sequences (Table S1). The genome sequence of *C. ramosus* is not yet available and hence degenerate primers were designed for the quantification of cda and chs transcripts using reference sequences of *Drosophila melanogaster* and *Anopheles darlingi* which are phylogenetically close species to *C. ramosus*. However, due to the non-availability of reference sequences for chitosanase in public domain, quantification of chitosanase transcripts was not possible. Undesiccated control values were set to 1 against which all other values were compared. The results were represented as fold induction relative to tubulin.

### Statistics

All experiments were carried out in three to eight replicates as appropriate (n = 30 larvae per replicate) under standard laboratory conditions. Wherever appropriate, mean ± SD values obtained were subjected to Student’s *t* test (comparison between two groups). One-way ANOVA was applied for comparisons between more groups and if mean values were found to be significantly different (*p* < 0.05), Student-Newman-Keuls post-hoc test was applied to analyse differences between the various desiccation and recovery time intervals. All statistical analyses were performed using Sigma Stat Version 4.0 (Systat Software, San Jose, CA).

## Electronic supplementary material

Supplementary movie 1

Supplementary material
