# Supplementary material for: High-throughput mass spectrometry analysis revealed a role for glucosamine in potentiating recovery following desiccation stress in Chironomus
Source: Sci Rep. 2017 Jun 16;7:3659. doi: 10.1038/s41598-017-03572-5 (PMC5473918; doi:10.1038/s41598-017-03572-5)
Supplement: Supplementary file 2 — Supplementary material [file 41598_2017_3572_MOESM2_ESM.pdf]

Supplementary Materials for

High-throughput mass spectrometry analysis revealed a role for glucosamine in potentiating recovery following desiccation stress in *Chironomus*

Leena Thorat[1,4], Dasharath Oulkar[2], Kaushik Banerjee[2], Sushama M. Gaikwad[3] and Bimalendu B. Nath[1*]

[1]Stress Biology Research Laboratory, Department of Zoology, Savitribai Phule Pune University, Pune-411007, India

[2]National Referral Laboratory, National Research Centre for Grapes, Pune-412307, India

[3]Division of Biochemical Sciences, National Chemical Laboratory, Pune-411008, India

[*]**Corresponding author:** Dr. B. B. Nath

Professor, Stress Biology Research Laboratory

Department of Zoology, Savitribai Phule Pune University,

Pune-411007, India.

E-mail: bbnath@gmail.com

Tel: +91-20-25601436 Fax: +91-20-25690617

[4]**Current address:** Department of Biotechnology, Savitribai Phule Pune University,

Pune-411007, India.

This PDF file includes:

Figs.S1 to S10
Tables S1 to S4
Movies S1

Supplementary Figures

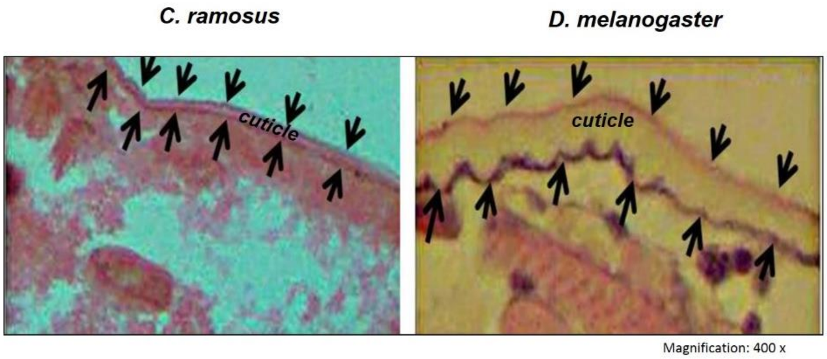

Fig. S1. Comparative view of the cross-sectional histology of the outer body integument of *C. ramosus* and *D. melanogaster larva*.

Histology- brief protocol: Bouin's fixed larvae were treated for serial dehydration in alcohols grades and paraffin embedding. Sections were deparaffinized in xylene and rehydrated in alcohol grades. Hematoxylin-eosin stained sections were examined under bright field microscope.

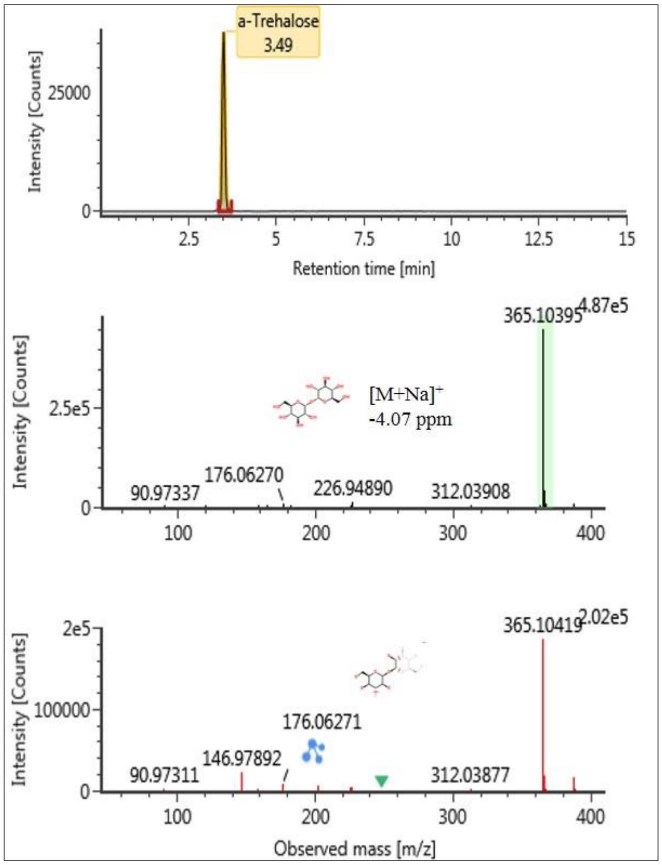

Fig. S2. Confirmation of uptake of trehalose by larvae upon trehalose feeding prior to desiccation exposure. Representative UPLC-QToF-MS chromatogram indicating the presence of trehalose in larvae fed with trehalose prior to desiccation. Feeding was carried out for 1 h and larvae were sacrificed 5 min post feeding to confirm the uptake of trehalose. We confirmed that the high peak signal is majorly due to exogenously fed trehalose (and to some extent due to basal endogenous trehalose levels) as compared to the corresponding low trehalose signal in the undesiccated control (Fig. 2B in the main manuscript). Trehalose was detected as [M+Na]+ adduct with mass accuracy -0.43 ppm with confirmatory ions (203.05248 Da, 145.04906 Da and 85.02768 Da).

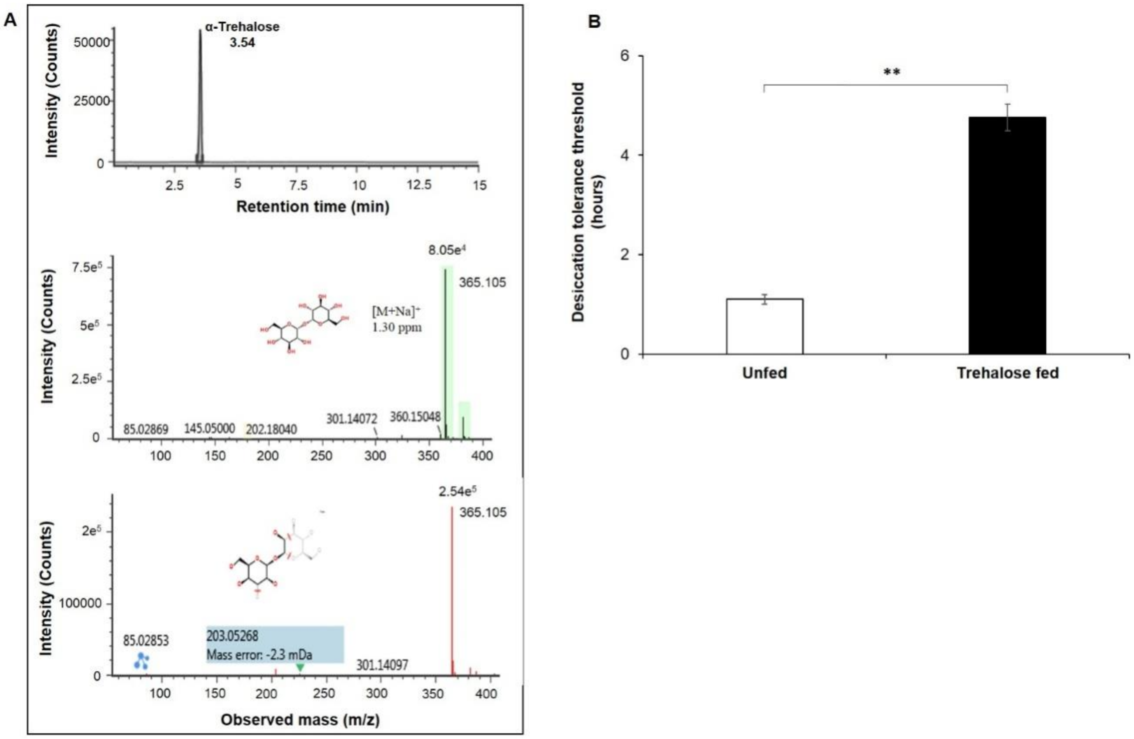

Fig. S3. Effects of trehalose feeding to the larvae feeding prior to desiccation (A) Representative UPLC-QToF-MS chromatogram indicating the presence of trehalose at the end of 50 min desiccation in larvae which were fed with trehalose prior to desiccation. The exceptionally high signal (nearly 8-fold excess compared to the unfed group) indicated the accumulation of exogenously fed + endogenous trehalose. Trehalose was detected as $[M+Na]^+$ adduct with mass accuracy -0.43 ppm with confirmatory ions (203.05248 Da, 145.04906 Da and 85.02768 Da). **(B)**Trehalose fed larvae exhibited increased endurance to desiccation (resulting in higher threshold for tolerance). Data represented are mean ± SD of three replicates. $^{**}p < 0.05$, Student's *t*-test.

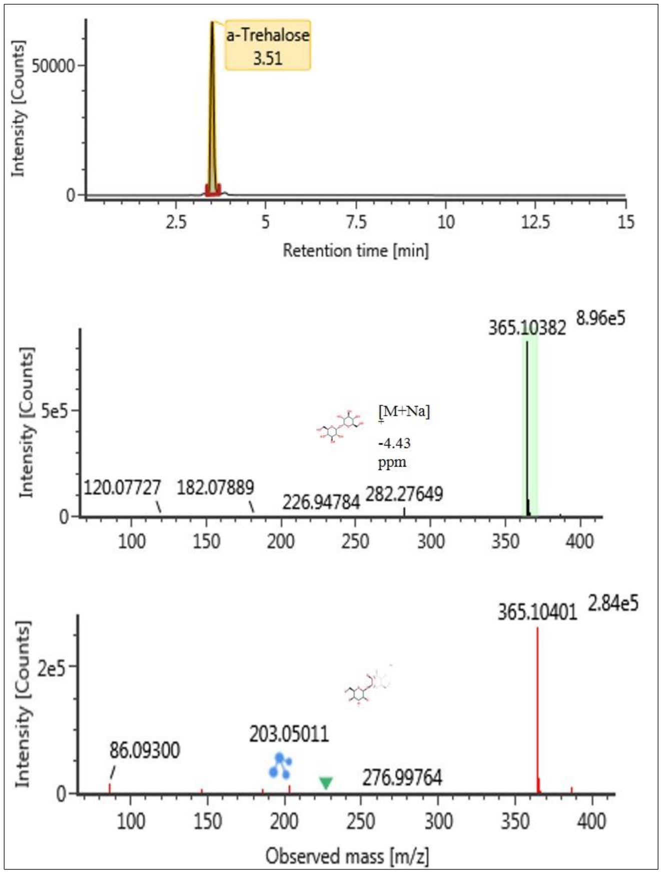

Fig. S4. Confirmation of uptake of trehalose by larvae upon trehalose feeding prior to rehydration. Representative UPLC-QToF-MS chromatogram indicating the presence of trehalose in trehalose-fed larvae prior to rehydration. Feeding was carried out for 1 h and larvae were sacrificed 5 min post feeding to confirm the uptake of trehalose. The considerably high peak signal is attributed to exogenously fed trehalose + endogenously accumulated trehalose levels during desiccation. Trehalose was detected as [M+Na]+ adduct with mass accuracy -0.43 ppm with confirmatory ions (203.05248 Da, 145.04906 Da and 85.02768 Da).

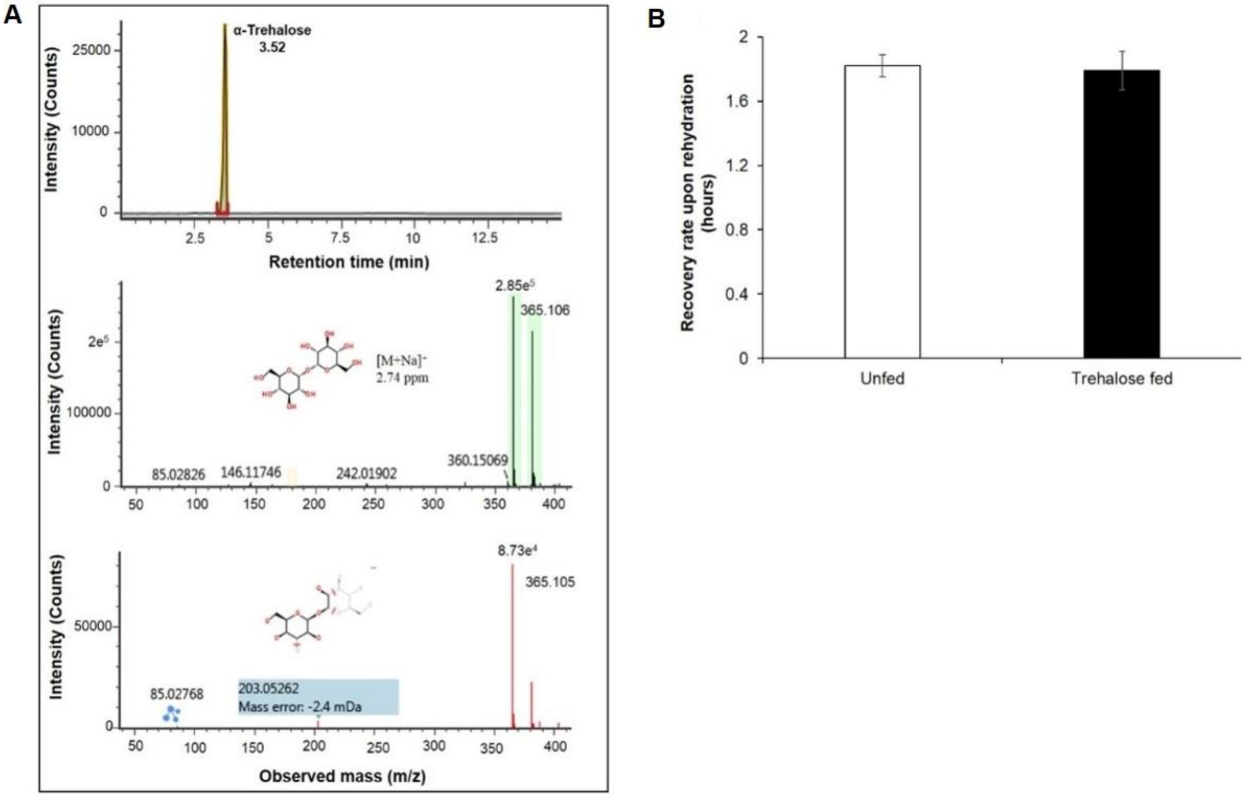

Fig. S5. Effects of trehalose feeding on larval recovery in larvae fed with trehalose prior to rehydration. **(**A**)** Representative UPLC-QToF-MS chromatogram indicating the presence of trehalose in 2 h rehydrated larvae that were fed with trehalose prior to rehydration. The low trehalose signal intensity compared to the desiccated larvae (Fig. 2C in main manuscript) reflected the hydrolysis of trehalose during rehydration. Trehalose was detected as $[M+Na]^+$ adduct with mass accuracy -0.43 ppm with confirmatory ions (203.05248 Da, 145.04906 Da and 85.02768 Da). **(**B**)** Larvae fed with trehalose prior to rehydration did not show significant improvement in recovery in comparison to the unfed larvae ($p > 0.05$; Student's *t*-test). Data represented are mean ± SD of three replicates.

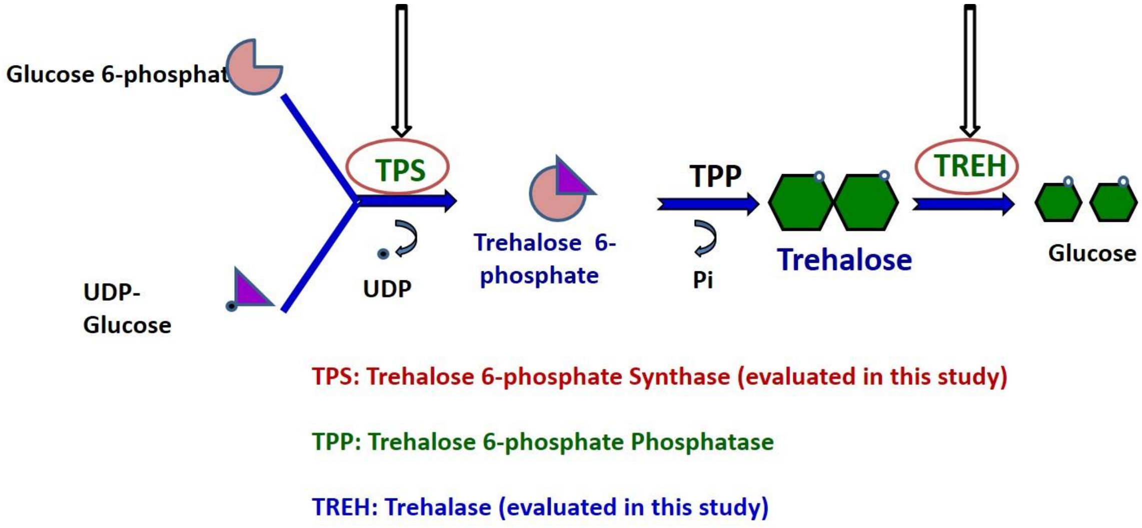

Fig. S6. The insect trehalose metabolic pathway. Insect trehalose synthesis is governed by trehalose 6-phosphate synthase (TPS) while trehalose (TREH) is responsible for its degradation.

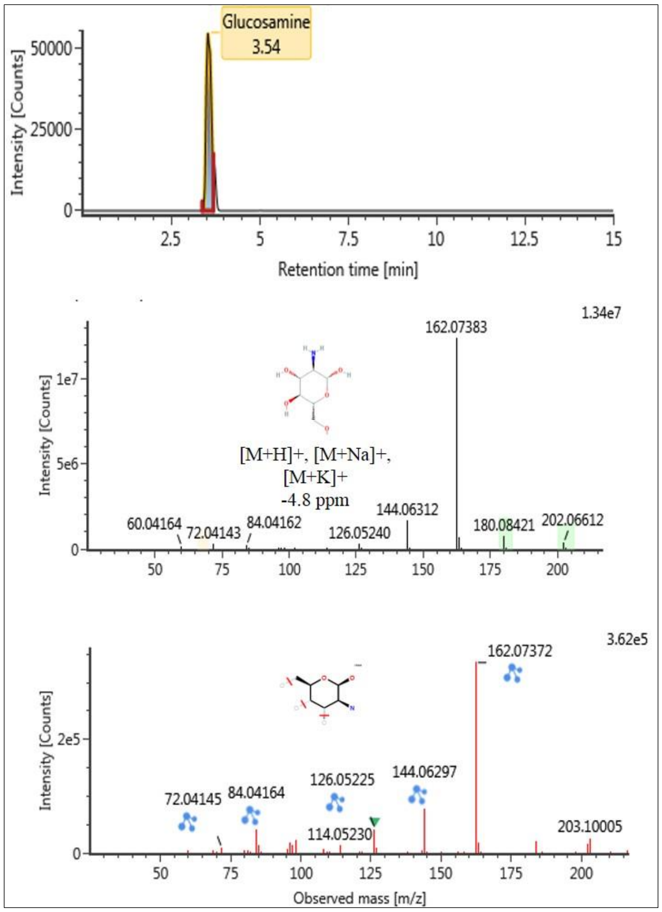

Fig. S7. Confirmation of uptake of glucosamine by larvae upon GlcN feeding prior to rehydration. Representative UPLC-QToF-MS chromatogram indicating the presence of GlcN in GlcN-fed larvae prior to rehydration. Feeding was carried out for 1 h and larvae were sacrificed 5 min post feeding to confirm the uptake of GlcN. The considerably high GlcN peak signal is attributed to exogenously fed GlcN as compared to the undesiccated control and desiccated group (Fig. 3B,C in the main manuscript). GlcN was detected as [M+H]+, [M+Na]+, [M+K]+. Of these, [M+Na]+ was prominent with mass accuracy 0.45 ppm along with confirmatory ions (162.07532 Da, 126.05370 Da and 84.04294 Da).

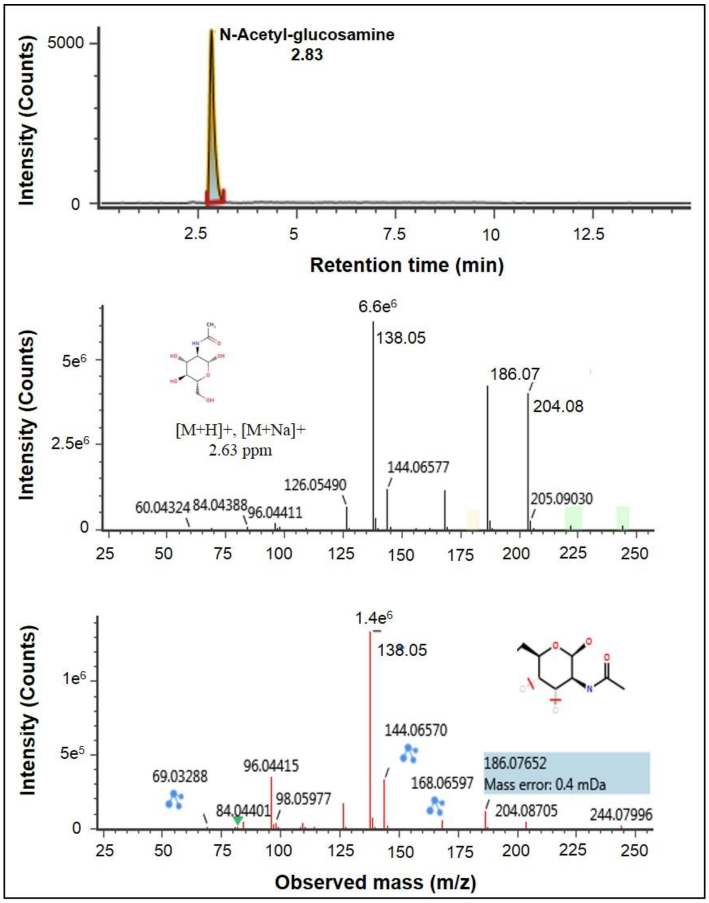

Fig. S8. UPLC-QToF-MS analysis depicting the chromatogram of standard of N-acetyl Glucosamine solution. Representative chromatogram indicating N-acetyl Glucosamine signal in the standard solution. N-acetyl Glucosamine was detected as $[M+H]^+$, $[M+Na]^+$ with mass accuracy 2.63 ppm along with confirmatory ions (138.05 Da, 144.06 Da and 186.07 Da). None of the experimental samples showed the presence of N-acetyl Glucosamine.

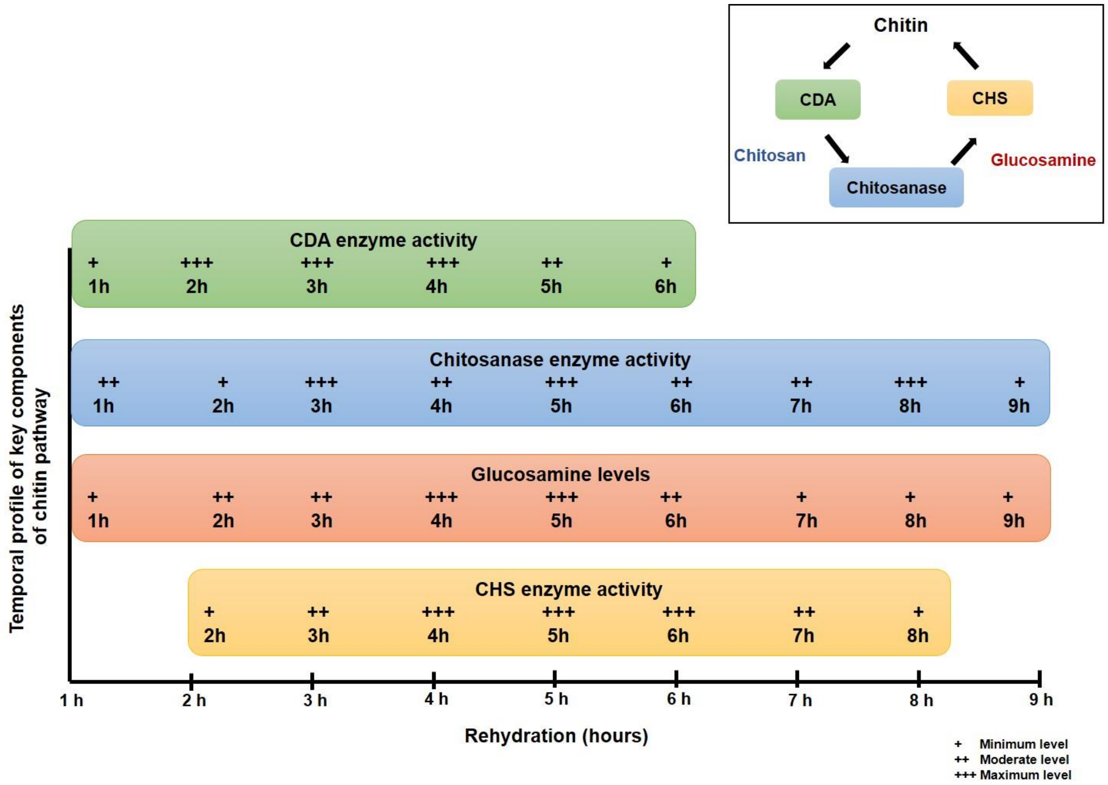

Fig. S9. Schematic depicting the sequence of events in the chitin pathway during rehydration. As evident, increment in the CDA activity began from 1h rehydration and continued to rise uptil 3h and maintained a relatively high level until 5h, thereafter declining to basal levels. CDA is responsible for the degradation of chitin into chitosan which is further used as substrate by chitosanase enzyme for the generation of glucosamine. Increasing CDA levels triggered subsequent rise in chitosanase enzyme activity up to 8h rehydration with intermittent increment and decrement in the activity. This resulted in the subsequent rise in glucosamine levels. Increase in chitosanase activity led to an almost concomitant rise in CHS enzyme which utilises glucosamine for chitin synthesis. Rise in CHS activity was substantially high during 3 and 6h rehydration. Simultaneously, beyond 5h rehydration, glucosamine levels started declining, indicative of the recruitment of glucosamine by CHS.

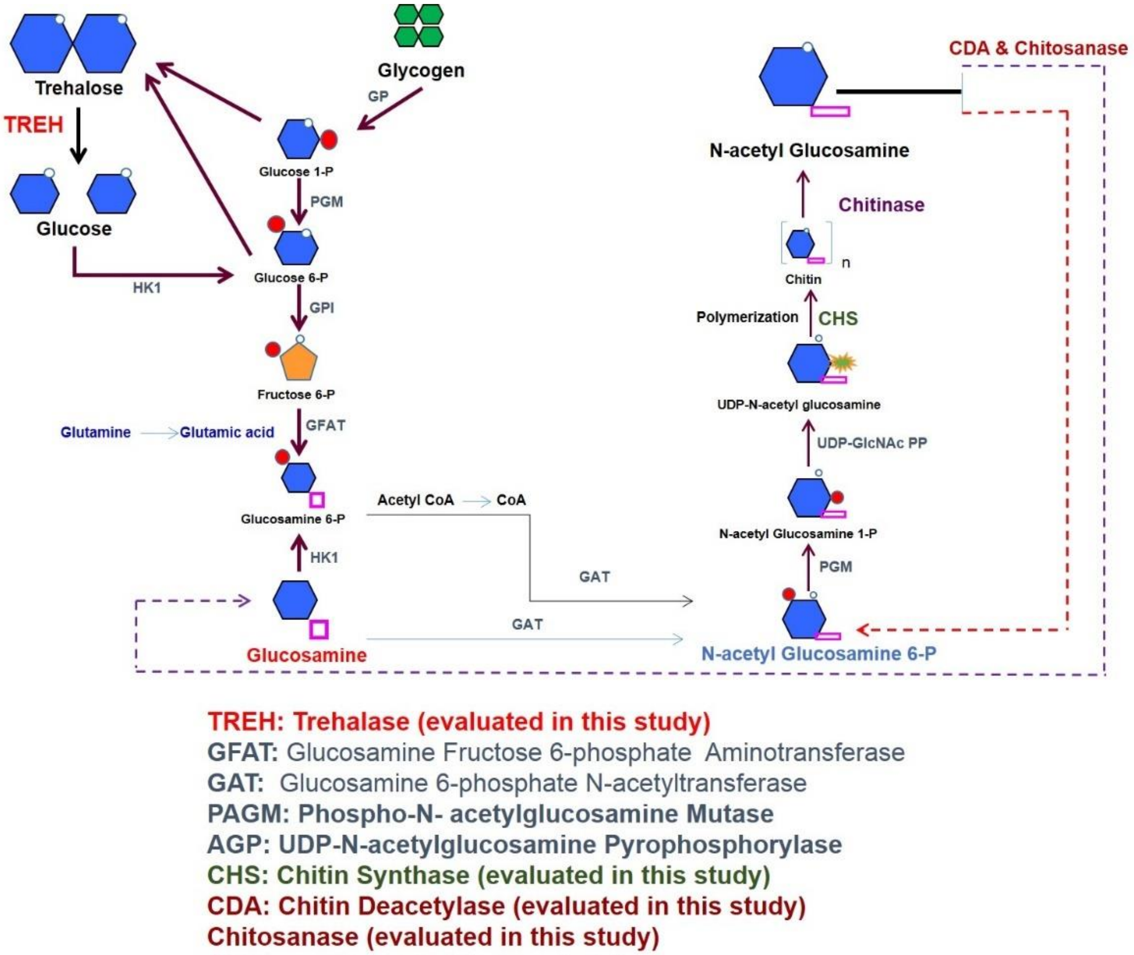

Fig. S10. The insect chitin metabolic pathway. Insect chitin synthesis essentially begins from trehalose. Glucosamine generated by trehalose hydrolysis is utilized for the synthesis of glucosamine 6-phosphate following a series of steps. Glucosamine 6-phosphate is then converted to N-acetyl glucosamine 6-P. Alternately, N-acetyl glucosamine 6-P can also be generated from glucosamine. After few more steps in the pathway, finally, polymerization of N-acetyl glucosamine produces chitin by CHS. Chitin can be degraded either by chitinase that releases free N-acetyl glucosamine monomers or by CDA which yields free glucosamine. The free glucosamine is amenable to the recycling process from where it can be again incorporated into the pathway.

<u>Table S1</u>:UPLC-(ESI)-QToF-MS data for α Trehalose ($C_{12}H_{22}O_{11}$)

Description	Expected mass (Da)	Observed mass (Da)	Mass error (ppm)	RT (min)	Calculated value (μg/individual)	Adducts	Fragment ions (Da)
Fig. 2A- *standard*	342.1162	365.1053	-0.43	3.56	500 ppm-trehalose standard solution	+Na	203.05248, 145.04906 and 85.0268
Fig. 2B- *undesiccated control*	342.1162	365.1052	-0.62	3.56	0.5 ± 0.016	+Na, +K	
Fig. 2C- *50 minutes desiccation without trehalose feeding*	342.1162	365.1049	-1.45	3.56	2.59±0.013		
Fig. 2D- *5 hours rehydration without trehalose feeding*	342.1162	365.1056	0.36	3.56	0.68±0.017		
Fig. S2 *Confirmation of trehalose uptake prior to desiccation in trehalose-fed larvae*	342.1162	365.1039	-4.07	3.49	3.66±0.09		
Fig. S3A- *50 minutes desiccation with trehalose feeding*	342.1162	365.1059	1.30	3.54	4.86±0.01		
Fig. S4 *Confirmation of trehalose uptake prior to rehydration in trehalose-fed larvae*	342.1162	365.1038	-4.43	3.51	7.9±0.012		
Fig. S5A- *2 hours rehydration with trehalose feeding feeding*	342.1162	365.1064	2.74	3.52	1.45±0.017		

Table S2: **UPLC-(ESI)-QToF-MS data for D-Glucosamine ($C_6H_{13}NO_5$)**

<table>
<tr><th>Description</th><th>Expected mass (Da)</th><th>Observed mass (Da)</th><th>Mass error (ppm)</th><th>RT (min)</th><th>Calculated value (µg/individual)</th><th>Adducts</th><th>Fragment ions (Da)</th></tr>
<tr><td>Fig. 3A- standard</td><td>179.0794</td><td>202.0687</td><td>-0.45</td><td>3.70</td><td>500 ppm- glucosamine standard solution</td><td rowspan="6">+Na, +H, +K</td><td rowspan="6">162.07532, 126.05370 and 84.04294 Da</td></tr>
<tr><td>Fig. 3B- undesiccated control</td><td>179.0794</td><td>202.0678</td><td>-3.8</td><td>3.75</td><td>1.25 ± 0.07</td></tr>
<tr><td>Fig. 3C- 50 minutes desiccation</td><td>179.0794</td><td>202.0678</td><td>-4.08</td><td>3.75</td><td>0.25±0.09</td></tr>
<tr><td>Fig. 3D- 6 hours rehydration without glucosamine feeding</td><td>179.0794</td><td>202.0671</td><td>-7.16</td><td>3.74</td><td>5.94±0.16</td></tr>
<tr><td>Fig. 3F- 6 hours rehydration with glucosamine feeding</td><td>179.0794</td><td>202.0677</td><td>-3.82</td><td>3.75</td><td>8.63±0.32</td></tr>
<tr><td>Fig. S7 Confirmation of glucosamine uptake prior to rehydration in GlcN-fed larvae</td><td>179.0794</td><td>202.06863</td><td>-4.8</td><td>3.53</td><td>4.79±0.27</td></tr>
</table>

<u>Table S3:</u> UPLC-(ESI)-QToF-MS data for standard solution of N-acetyl glucosamine($C_8H_{15}NO_6$)

**Description**	**Expected mass (Da)**	**Observed mass Da)**	**Mass error (ppm)**	**RT (min)**	**Adducts**	**Fragment ions (Da)**	**Calculated concentration**
Fig. S3-<br>*standard*	221.0899	222.0978	2.63	2.83	+H, +Na	138.05, 144.06	1000.00ppm- N-acetyl glucosamine standard solution

<u>Table S4:</u> List of Primers for RTqPCR

**No.**	**Oligoname**	**Sequence (from 5' to 3')**
1.	Tubulin_forward	GCGTGAATGTATCTCAGTTCATGTTG
	Tubulin_reverse	GACGTGCTTTCCAGCACCAGT
2.	Tps_forward	GTCCGTGGACGATTTCGAT
	Tps_reverse	AAGGTTCCGTCGTAGTCCAG
3.	Treh_forward	CGAGGCCAAGAATATGTCACT
	Treh _reverse	CGAACTCATCGGCGTTGTA
4.	Cda_forward	TGCAGGATAAGGGCTACGAG
	Cda_reverse	CCTTGTATTGGGTGTTGCGG
5.	Chs_forward	TGTGCACCCTGTTGTTGAAG
	Chs_reverse	AGATGTTGGCAATGGTGGAC

Movie S1

Time-lapsed video demonstrating the desiccation and revival of a single larva of *C. ramosus.* The first 30 s of the video shows actively moving larva in water (undesiccated control). Next, the larva was transferred to Whatman filter paper to remove excess moisture adhering to the body. The larva was then gently picked up using a non-abrasive brush and transferred to a Petri dish lined with dry tissue paper and kept in the desiccator. 10 µl of water was given to the larva in order to prevent drying during the process of placing the larva inside the desiccator. Larva was then allowed to dehydrate inside the desiccator. Threshold desiccation tolerance of *C. ramosus* was 50±10 min. In this video, the larva was desiccated for 40 min. Thereafter, the desiccated larva was gently transferred using a brush and allowed to rehydrate in water. During the first 15-20 min of rehydration, larval body 'jerks' were observed followed by progressive signs of revival (increase frequency of wriggling) over the next 2h of rehydration min with active body movements. Initial slight body movements characterized by 'jerks' and 'wriggling' were considered as the early signs of revival. Larval survival was judged by gentle stimulation with a brush. With progressive rehydration, larval movements became more prominent and were now characterised by increased frequency of wriggling movements defined as 'undulatory movement' which was used as a behavioural parameter for judging larval recovery after desiccation stress. One larval undulation was considered as one complete wave-like motion, starting from the head and traversing in an antero-posterior direction which ends at the tip of the posterior parapods. We defined the beginning of this undulatory movement as larval recovery (details furnished in materials and methods).
